# The Hydraulic Mechanism of the Unfolding of Hind Wings in *Dorcus titanus platymelus* (Order: Coleoptera)

**DOI:** 10.3390/ijms15046009

**Published:** 2014-04-09

**Authors:** Jiyu Sun, Mingze Ling, Wei Wu, Bharat Bhushan, Jin Tong

**Affiliations:** 1 Key Laboratory of Bionic Engineering (Ministry of Education), Jilin University, Changchun 130025, China; E-Mails: lingmingzeowen@sohu.com (M.L.); weiwu13@mails.jlu.edu.cn (W.W.); jtong@jlu.edu.cn (J.T.); 2 Nanoprobe Laboratory for Bio- & Nanotechnology and Biomimetics (NLB^2^), the Ohio State University, 201 W. 19th Avenue, Columbus, OH 43210-1142, USA

**Keywords:** beetle, hind wing, unfolding, hydraulic mechanism, micro air vehicles (MAVs)

## Abstract

In most beetles, the hind wings are thin and fragile; when at rest, they are held over the back of the beetle. When the hind wing unfolds, it provides the necessary aerodynamic forces for flight. In this paper, we investigate the hydraulic mechanism of the unfolding process of the hind wings in *Dorcus titanus platymelus* (Oder: Coleoptera). The wing unfolding process of *Dorcus titanus platymelus* was examined using high speed camera sequences (400 frames/s), and the hydraulic pressure in the veins was measured with a biological pressure sensor and dynamic signal acquisition and analysis (DSA) during the expansion process. We found that the total time for the release of hydraulic pressure during wing folding is longer than the time required for unfolding. The pressure is proportional to the length of the wings and the body mass of the beetle. A retinal camera was used to investigate the fluid direction. We found that the peak pressures correspond to two main cross-folding joint expansions in the hind wing. These observations strongly suggest that blood pressure facilitates the extension of hind wings during unfolding.

## 1. Introduction

Insects of certain orders, such as Dermaptera, Coleoptera, and some insects of Blattodea, require large, long hind wings to provide the necessary aerodynamic forces for flight [1–5]. Because hind wings are vulnerable to damage, they are folded under the forewings for protection. Forbes [6] first discussed the folding of beetle hind wings. When beetles are at rest, the wings are held over their back, which may involve both longitudinal and transverse folding of the wing membrane. Folding occurs along the flexion lines [7]. Although the fold lines of the hind wings of beetles are transverse, the fold lines are typically radial to the base of the wing, allowing an inward retraction of the wing tip, and the adjacent sections of the wing are folded over or under each other [4,8]. The beetle hind wing is comprised of a combination of several basic mechanisms consisting of four plates connected by hinges [9], and the system possesses a single kinematic degree of freedom [10]. Some of the flexion lines are active not only while the wing is folded away after flight but also during the stroke, where flexion lines play a dynamic role in altering the wing profile. Wing venation also affects the folding patterns [11–13]. In general, wing extension most likely results from the contraction of the muscles attached to the basalar sclerite or, in some insects, to the subalar sclerite [14].

Energy is required to unfold and/or fold the hind wing and to prevent wear at critical locations in the wing [15]. This energy requirement has major mechanical and geometrical implications. The muscles do not extend beyond the base of the wing, and transverse folding occurs along the length of the wing [9]. The mechanism of the unfolding process of the wing could result from an increase in blood pressure. The increased blood pressure may straighten the costal margin to an extent; at this point, it has been suggested that hydraulic mechanisms for unfolding activate [16,17], but this mechanism has not been confirmed [11,18,19]. A study of the folding and unfolding of hind wings to understand the relationship between the flight behavior of insects and the mechanisms that control such flight revealed that the wings of beetles flex appreciably along fold creases during flight [19]. Aerodynamic and inertial forces may play a role in the extension of wings. Because there are no muscles in the beetle hind wing, the control of folding and unfolding is remote and may be achieved in one of four ways: (1) by leverage, storage and release of elastic energy; (2) by the action of other body structures [9]; (3) by promoting the mechanism of unfolding along with hydraulics [11,16–19]; (4) and in some insects of the order of Coleoptera by folding of the abdomen [2,16,20]. Occurrence of resilin, a rubber-like protein, in some mobile joints has multiple functions. Resilin provides elasticity, allowing the wing to be deformable by aerodynamic forces that may result in elastic energy storage in the wing. Therefore, the distribution pattern of resilin in the wing correlates with the particular folding pattern of the wing; resilin is found in places where extra elasticity is needed [21,22].

In this paper we investigate the hydraulic mechanism of the unfolding of hind wings in *Dorcus titanus platymelus* (Oder: Coleoptera). This work provides insight into the flight behavior of insects. The folding/unfolding mechanisms of the beetle hind wing may provide design inspiration for portable micro air vehicles (MAVs) with morphing wings and bioinspired deployable systems.

## 2. Results and Discussion

**Figure 1.**
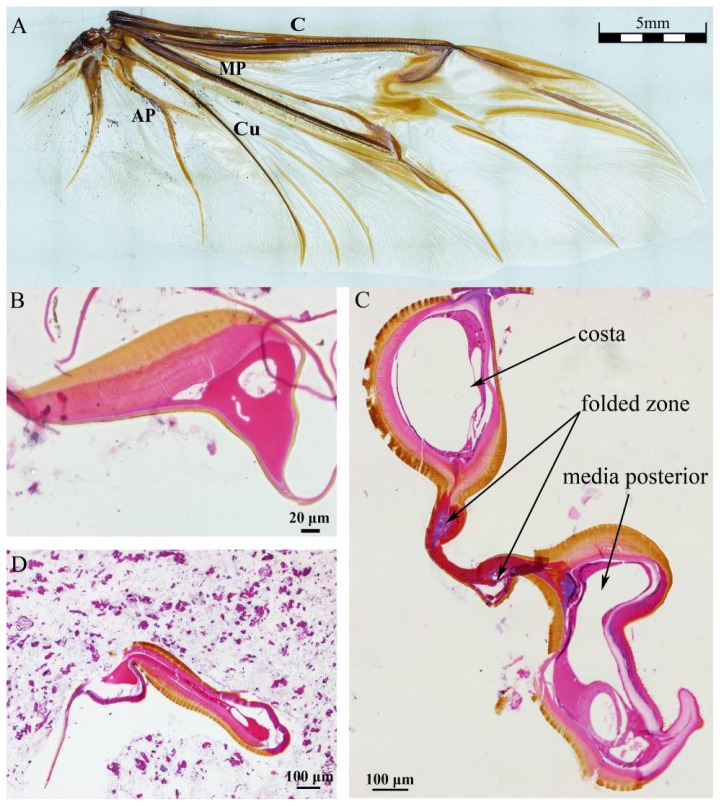
(**A**) shows the venation of hind wing of *Dorcus titanus platymelus*; The cross sections of (**B**) the root, (**C**) the folded zone and (**D**) the tail of costa (40×) by inverted fluorescence microscope where C is costa, MP is media posterior, Cu is cubitus, and AP is anal posterior. The vein cavity is regularly.

Figure 1A shows the venation of hind wing of *Dorcus titanus platymelus*. Figure 1B–D shows the cross sections of the root, the folded zone and the tail of costa (40×), respectively. The exocuticle, or outer layer, appears brown, and the endocuticle and epidermis layers appear light pink (the thin basement membrane). Because the cavities of the veins are connected with hemocoel, hemolymph can flow into the wings [14]. The figure shows an irregularly shaped vein cavity, and its cross sectional area reaches a maximum in the folded zone. Both ends of wire-like objects are wing membrane. Figure 1B shows that the costa, the upper exocuticle and endocuticle layer are thicker toward the top than at the bottom of the wing, which may be related to flight mechanics performance. The upper cavity in Figure 1C is the costa cavity, the middle is folded zone, and the lower cavity is the media posterior vein cavity. Only two layers of the epidermal layer folding area are clearly visible; we suspect that blood hemolymph in the vein cavity was lost during the slicing/preparation process, so it appears as a cavity in the image below. The vein cavity area in the tail is the smallest (Figure 1D), shown as narrow strip. The vein cavity shape is irregular, and the cross-sectional area along the costa is variable, bearing the characteristics of flight performance.

**Figure 2.**
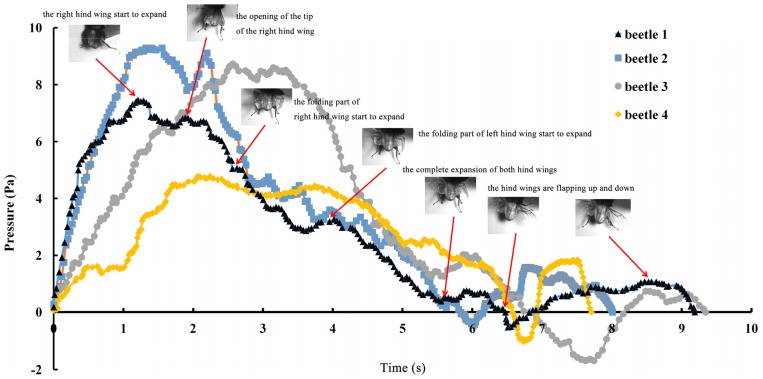
The change in blood pressure in the veins of the hind wings with time. The photos captured with a high-speed camera show the relationship of the unfolding actions of the hind wings with fluctuations in blood pressure.

The series of unfolding movements of the hind wings of four beetles are shown in Figure 2 at 0.62 s, the right elytron was raised; at 0.82 s, the double elytra lifted to a sufficient height and angle resulting in a display of gradually rotating hind wings. Because the right hind wing was stacked on top of the left hind wing, the right hind wing was extended first. At 1.17 s, the unfolding action commenced; at 5.51 s, dual hind wings were fully extended, and the beetle could fly. Although the left and right hind wings were obviously not expanded at the same time, the hind wings beat simultaneously to enable flight to take place. Thus, there is an automatic mechanism that promotes the hind wings to beat synchronously. Previous authors proposed a resting state in which the hind wing automatically returns when the elytra are lifted or when the wing is isolated by dissection. However, these authors assumed that the underlying mechanism was based on the intrinsic elasticity of the wing and not on the activity of the thoracic muscles [4]. On the contrary, the full recovery of folded hind wings took a little longer (~6 s) than the time required for unfolding.

**Figure 3.**
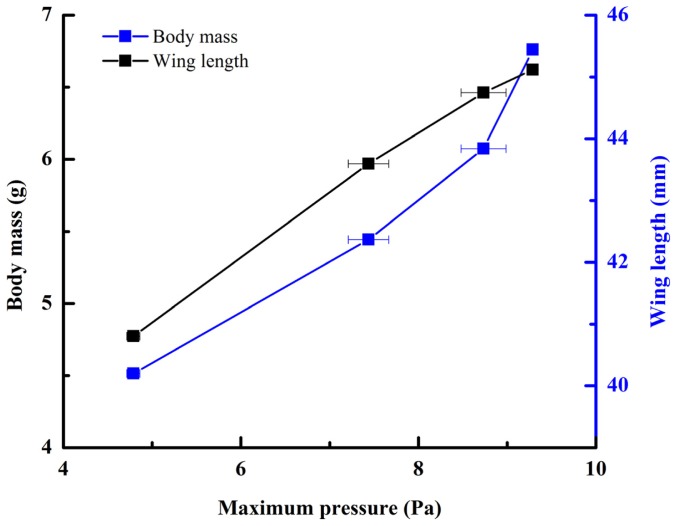
Pressure is proportional to the length of wings and the body mass.

The pressure measurements obtained in the beetle hind wings (Figure 2, averaging every 120 points) showed a peak in maximum pressure (7.46 Pa) at 1.17 s, which allowed the right hind wing to expand. At 1.85 s the second peak in pressure appeared (6.82 Pa) corresponding to the opening of the tip of the right hind wing. The complete expansion of both hind wings, at 5.51 s corresponded to a pressure is 0.43 Pa, followed by a slow release to zero pressure which is completed in 6.47 s. After this point when the hind wings are flapping up and down, the pressure values indicated some intrinsic elasticity in the wing due to hydraulic energy, which can make flight kinematics more stable. The subsequent small peaks correspond to fluctuations in blood pressure to overcome the remaining folds. Using the same method with 3 additional beetles in a comparison test, only two peaks were found during unfolding, but the maximum pressure varied between 6.07 and 9.29 Pa. The time interval between the two peaks ranged from 0.68 to 1.59 s, and the total time of expansion ranged from 5.8 to 6.73 s. Pressure is proportional to the length of wings and the body mass of the beetle (Figure 3). We can deduce that blood pressure in the veins of the hind wings increased until overcame the spring mechanism of resilin, at which point the hind wings straighten. A limitation of this methodology is the difficultly to steadily fix a sensor on the vein to take consistent measurements due to the wing folding process combined with elytra closing the wing plume.

**Figure 4.**
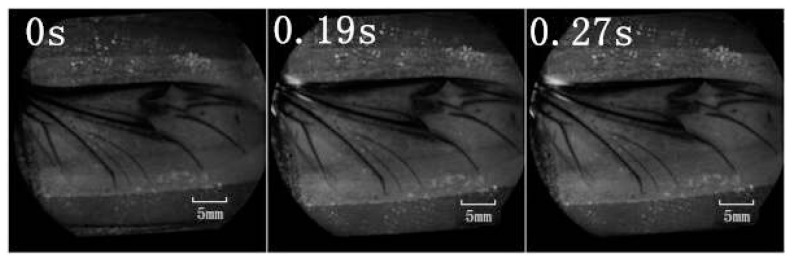

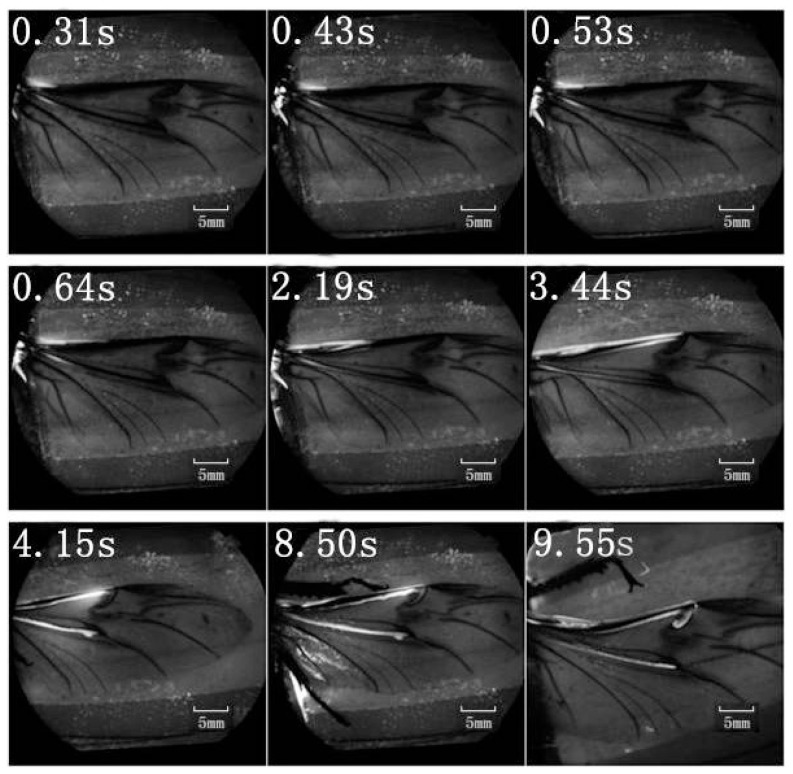
Fluorescence flow sequence of wing unfolding in *Dorcus titanus platymelus*.

A retinal camera was used to confirm which veins were involved in the hydraulic mechanism of the unfolding of hind wings. The experimental live beetle was injected in the abdomen with a fluorescent agent. Four seconds later, fluorescence was observed in the costa and media posterior (Figure 4). The fluorescence stopped after 9.55 s. This value is larger than that measured in Figure 2 (~6 s). One possible explanation is that the hind wing in this test (Figure 4) was fully expanded and fixed; on the contrary, the beetle in the pressure measurement (Figure 2) was able to move freely accelerating blood flow. Fluorescence in the costa stopped at the marginal joint and then flowed to the humeral cross-vein. Fluorescence in the media posterior stopped at the intersection of the folding area, the transverse fold line, even though the beetle was still alive (Figure 4). We observed that the flow of body fluids is limited to movement within the main veins—the costa and media posterior—and stops at the folding region. This phenomenon is important to our subsequent analysis of body fluids in the folding and unfolding of hind wings.

**Figure 5.**
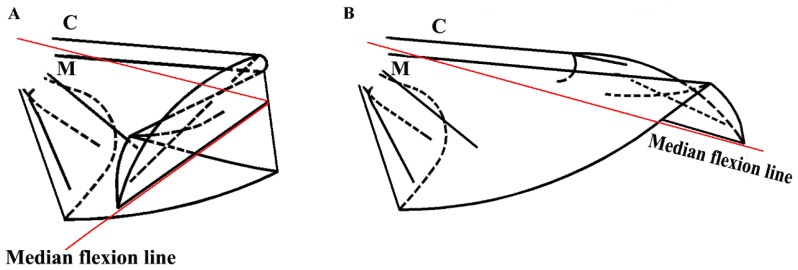
Schematic representation of the unfolding mechanism of the hind wings of *Dorcus titanus platymelus*. (**A**) Fully opened wing; and (**B**) Folded wing. C, costa; M, media posterior.

The costa is hollow, as shown in Figure 1, but the fluorescence shown in Figure 4 did not progress; an explanation for this finding could be the viscosity resistance in the veins and the hydraulic thrust force. Blood is a viscoelastic fluid and its rheological properties, viscosity and elasticity, depend on the rate of flow or shear rate. We found that the two peak pressures correspond to two main cross-folding joint expansions in the hind wing (Figure 5A,B). This is facilitated by a series of flexion lines running longitudinally, transversely, and obliquely across the wing surface [1,2,17,19]. If unfolding is driven by muscles, then promotion might be driven by a mechanism utilizing intrinsic elasticity [4]. We suggest that the hydraulic mechanism of blood pressure overcomes the spring mechanism in the wing structure at the cross-folding joint. This process may be aided by movement in the abdomen to keep the wing in folded position [2]. The hydraulic drive unfolding system has many advantages. The hydraulic system in the hind wings can be a multifunctional power source. The hind wings are considerably large relative to the beetle’s body size. Hydraulic pressure is also utilized in expanding and maintaining wings. Thus, insects of the order of Coleoptera have no requirement for a large mass of muscles and appendages to produce mechanical energy. Small muscles in the abdomen control hydraulic pressure, which generates a high output of power. Due to the limited muscle mass needed to control the hydraulic folding/unfolding system in beetle hind wings, this wing structure is greatly simplified as compared with the folding/unfolding of bird wings. The hydraulic drive system allows an easy transfer of energy over long distances for expanding of the hind wings. Because the fluid properties of blood allows elastic energy storage and absorption shock, movement of beetle hind wings can be achieved through a simple and stable process.

This work provides insight into the flight behavior of insects; the folding/unfolding mechanisms of the beetle hind wing may provide design inspiration for portable MAVs with morphing wings [23]. By actuation of memory shape alloy wires, artificial wings can be unfolded to provide an actuation force at the wing base and along the leading edge of the vein [24]. Additionally, the beetle hind wing folding/unfolding behavior provides inspiration for optimized biomimetic deployable system design [10], such as flapping of a wing based on structural interaction with the fluid medium [25].

## 3. Specimens and Experimental Methods

*Dorcus titanus platymelus* is a type of phytophagous, aggressive beetle, with strong flight capability. The hind wings are folded beneath the elytra when not in use, and these beetles are capable of reducing the length of wings by up to 55%. Beetles were captured in the wild, in Shanghai, China. The microstructures of cross sections of veins were captured using an inverted fluorescence microscope (OLYMPUS, LX71, Olympus Optical Co., Ltd., Tokyo, Japan). After the hind wings were dehydrated, the hind wings were prepared for analysis by embedding, sectioning and staining (hematoxylin-eosin). Flying beetles were photographed with a high-speed camera (OLYMPUS, i-SPEED 3, camera speed of 400 frames/s, Olympus Corp., Tokyo, Japan).

**Figure 6.**
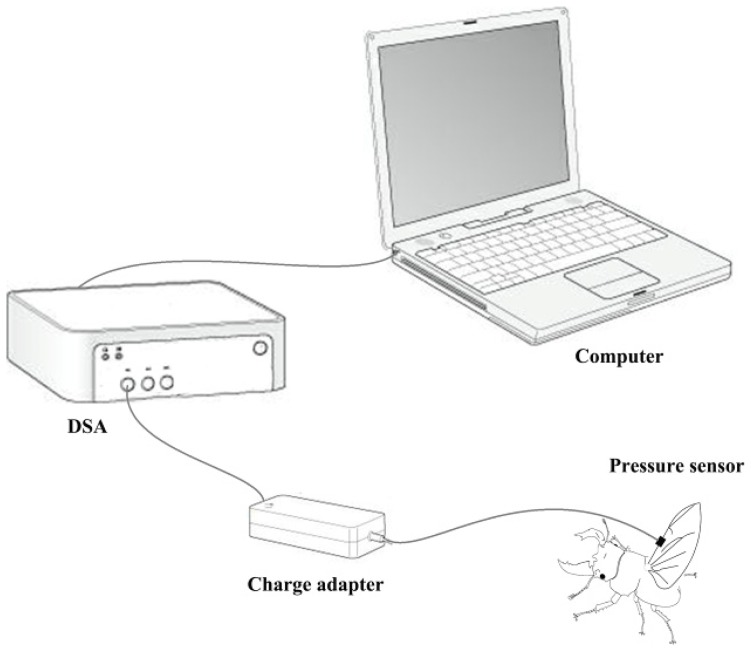
Schematic of the equipment used for measuring the change in blood pressure during expansion of wings.

A biological pressure sensor and dynamic signal acquisition and analysis (DSA) were designed as a control system to investigate variation of fluid pressure in the veins of the hind wings (Figure 6). The sensor was a polyvinylidene fluoride (PVDF) piezoelectricity thin-film sensor with a diameter of 5 mm, thickness of 30 μm, detection sensitivity (4 Hz) of 10^11^ *m* · Hz^1/2^/W, sound velocity of 2000 m/s, relative dielectric constant (ɛ/ɛ_0_) of 9.5 ± 1.0 (1 KHz), and electro-mechanical coupling factor (K_33_) of 10%–14%. The sensor was installed on the costa vein by double sides-sided adhesive tape, and the elytron on the testing side was removed but preserved on the opposite side. The beetle was kept alive and allowed to move freely. When the sensor and measurement system were completely installed, the experimenter used a stick to simulate the beetle’s abdomen. This resulted in the beetle expanding its hind wings at which point the data were collected. These tests were repeated four times. General piezoelectric materials are sensitive to pressure, when you stretch or bending a piece of PVDF piezoelectricity thin-film, the film between the upper and lower electrode surface can produce an electrical signal (charge or voltage), and is proportional to the strain of stretch or bending. In order to improve the signal-to-noise ratio of the analog input signal, the signal filter was used to attenuate the noise (set in charge adapter, Figure 6), removing unnecessary frequency (such as electric noise, interfering signal), and obtain certain frequency band signals. The relevant parameters can be obtained from preliminary experiments which need keep the hind wings inactive. Filter used in this test using the second-order active filter form: 0.8 Hz second-order high-pass filter (second-order Butterworth high pass filter) filter DC (direct-current) component; AC (alternating current)component after 200 times magnification input 48 Hz second-order low-pass filter (second-order Butterworth low-pass filter) remove the high frequency interference include noise cause by the power and the bending pressure change of sensor, and will maintain the signal between 0 and 5 V.

To confirm which veins of the hind wings were involved in the hydraulic mechanism during unfolding, a retinal camera (TOPCON, TRC-50DX-Type IA, Topcon Corp., Tokyo, Japan) was used. Retinal camera testing was performed with a fluorescent agent to color the walls of the veins. One milliliter of green fluorescent indicator (FITC) was injected in beetle abdomen at time 0 (Figure 4). The measurement was taken with an excitation wavelength of 488 nm in retinal camera.

## 4. Summary

The folding/unfolding characteristics of hind wings are special features of beetles. Folding may sometimes occur along flexion lines. In general, extension of beetle hind wings most likely results from the hydraulic pressure of blood.

In the current study, we demonstrated fluctuations of hydraulic pressure, appearing as two peaks during unfolding of wings; the first maximum peak corresponded to the initiation of unfolding, and the second peak corresponded to the unfolding of the tip of the wing. The maximum value of the peak in pressure varied between 6.07 and 9.29 Pa. The time interval between the two peaks ranged from 0.68 to 1.59 s, and the total time of expansion ranged from 5.80 to 6.73 s. Pressure is proportional to the length of wings and the body mass. The total time for the release of hydraulic pressure during folding of wings is longer than the time required for unfolding. In addition, we found that blood flows only through the costa vein, ending at the marginal joint which then turns towards the humeral cross-vein, and the media posterior veins, ending at a transverse fold. Our results demonstrate that blood pressure facilitates the extension of hind wings during the unfolding process. We anticipate our work to be the basis of further sophisticated research on the folding and unfolding mechanisms of beetle hind wings as these mechanisms may provide design insights for portable MAVs with morphing wings and give inspiration to the development bioinspired deployable systems.
